# Triggering Receptor Expressed on Myeloid Cells‐2 Regulates Innate Lymphoid Cell Levels in Bleomycin‐Induced Pulmonary Fibrosis

**DOI:** 10.1002/kjm2.70209

**Published:** 2026-04-02

**Authors:** Hsiao‐Chin Shen, Nien‐Jung Chen, Chuan‐Yen Sun, Wen‐Kuang Yu, Vincent Yi‐Fong Su, Wan‐Ling Yang, Wei‐Chih Chen, Kuang‐Yao Yang

**Affiliations:** ^1^ Department of Chest Medicine Taipei Veterans General Hospital Taipei Taiwan; ^2^ Institute of Microbiology and Immunology School of Life Sciences, National Yang Ming Chiao Tung University Taipei Taiwan; ^3^ School of Medicine, National Yang Ming Chiao Tung University Taipei Taiwan; ^4^ Division of Evidence‐Based Medicine, Department of Medical Education Taipei Veterans General Hospital Taipei Taiwan; ^5^ Department of Internal Medicine Taipei City Hospital Taipei Taiwan; ^6^ Taipei Hospital, Ministry of Health and Welfare Taipei Taiwan; ^7^ Institute of Emergency and Critical Care Medicine National Yang Ming Chiao Tung University Taipei Taiwan; ^8^ Cancer and Immunology Research Center National Yang Ming Chiao Tung University Taipei Taiwan

**Keywords:** human, immunity, innate, pulmonary fibrosis, TREM2 protein

## Abstract

Idiopathic pulmonary fibrosis, a pathological change causing poor outcomes, is not reversible despite current antifibrotic therapy. Emerging evidence suggests that innate lymphoid cells (ILCs) mediate lung inflammation and fibrosis after stimulation by endogenous factors. Triggering receptor expressed on myeloid cells‐2 (TREM2) is known to regulate inflammation in various diseases. However, the relationship between TREM2 and ILCs in pulmonary fibrosis is unclear. This study aimed to explore the role of TREM2 in regulating ILC activation in bleomycin (BLM)‐induced pulmonary fibrosis. We first examined the role of TREM2 in regulating ILC expression in a mouse model of BLM‐induced pulmonary fibrosis, and subsequently assessed the levels of ILCs, pulmonary fibrosis, and inflammation using histological staining and molecular biology techniques. These results were compared between wild‐type (WT) and TREM2 knockout (KO) mice. Compared with those in WT mice, inflammatory cell aggregation and collagen fiber deposition were more prominent in TREM2‐KO mice after intratracheal BLM injection. Compared with those in WT mice, there was a pronounced increase in GATA3 and RORγt expression in lung tissues from TREM2‐KO mice. In adaptive transfer experiments, ILC‐enriched population isolated from WT mice after BLM stimulation in vivo increased the expression of TGF‐β, α‐SMA, and collagen‐1 in naïve WT mice. Immunofluorescence staining revealed GATA3 expression in the lung tissues of TREM2‐KO mice after BLM stimulation. Compared with those receiving ILCs from WT mice, those receiving adoptive transfer of ILC‐enriched population from TREM2‐KO mice exhibited more lung injury and fibrosis. In conclusion, TREM2 decreases ILC activity to reduce BLM‐induced pulmonary fibrosis.

## 1 Introduction

Idiopathic pulmonary fibrosis (IPF) is a progressively worsening and devastating form of interstitial lung disease. The exact cause of IPF is not fully understood. However, several risk factors, such as smoking, aging, infections, and environmental exposures, are closely linked to IPF. These factors contribute to the progressive fibrosis of lung tissue [1]. Its incidence ranges from approximately 2.8 to 19 cases per 100,000 person‐years in Europe and North America, and the prognosis remains poor, with a 5‐year mortality rate of about 70% [2, 3]. Therefore, investigating new targets for the effective clinical treatment of IPF is crucial.

Innate lymphoid cells (ILCs) are classified into three primary groups on the basis of their resemblance to T helper cell subsets: ILC1, ILC2, and ILC3. They play key roles in the immune system: ILC1s combat intracellular pathogens (such as viruses) and target cancer cells by producing interferon‐gamma; ILC3s defend the gut against bacteria and fungi by secreting IL‐17 and IL‐22, thereby reinforcing intestinal immunity [4]; and ILC2s play a role in Type 2 inflammatory diseases, including asthma and atopic dermatitis. These cells produce Type 2 cytokines and interact with immune and nonimmune cell populations in local tissues [5, 6].

A recent study has shown that ILC2 is also involved in lung defense and repair [7]. In animal studies, ILC2s were found to contribute to worsened lung inflammation and fibrosis caused by bleomycin (BLM) [8] and even induce spontaneous lung fibrosis [9]. Additionally, a separate study noted that bronchoalveolar lavage fluid from patients with IPF contained higher ILC2 levels than did that from controls [10]. Furthermore, a study conducted on patients with systemic sclerosis revealed that elevated ILC2s in peripheral blood correlated with interstitial lung involvement [11]. However, growing evidence indicates that ILC3s also play a role in lung disease. These cells are widely distributed within the alveolar capillary beds, where inhaled pathogens first encounter the lung parenchyma [12]. IL‐22 produced by lung‐resident ILC3s preserves epithelial integrity and reduces inflammation [13]. Furthermore, ILC3s have been implicated in pulmonary fibrosis in both patients and animal models [14]. In fibrotic lung disease patients, ILC3‐derived IL‐17A levels are elevated, and in murine models IL‐17A drives neutrophil‐mediated lung fibrosis [15]. Together, these findings suggest that ILCs—particularly ILC2s and ILC3s—may contribute to the development of fibrosis.

Triggering receptor expressed on myeloid cells‐2 (TREM2), a member of the myeloid trigger receptor family, is expressed in the brain, lungs, bone, and other tissues [16]. This molecule mitigates inflammation by suppressing Toll‐like receptor expression and macrophage activation [17]. Numerous studies have associated TREM2 with various lung conditions. Research has revealed increased levels of TREM2 and M2 macrophage markers in individuals with chronic obstructive pulmonary disease (COPD) compared with those in good health. Furthermore, those with COPD exhibiting high TREM2 levels often have diminished lung function [18]. In cases of pulmonary fibrosis, a notable increase in TREM2^+^ CD206^+^ macrophages is triggered by severe acute respiratory syndrome coronavirus 2‐induced pulmonary fibrosis [19]. Additionally, higher levels of TREM2 were found in IPF patients and in a mouse model of BLM‐induced pulmonary fibrosis [20]. In contrast, some studies suggest that TREM2 protects against lung injury and pulmonary fibrosis. Animal studies have revealed that mice devoid of TREM2 display increased lung inflammation following bacterial exposure [21]. Similar observations have been made in lipopolysaccharide‐induced acute lung injury cases, where TREM2 knockout (KO) mice show intensified lung damage and greater neutrophil infiltration in their lung tissues [22]. Another study revealed that promoting TREM2 expression could attenuate lung injury in patients with lung ischemia–reperfusion injury [23]. A recent study reported that blocking TREM2 alleviates pulmonary fibrosis by regulating lipid metabolism [24] and alveolar macrophages [25].

Although numerous studies have reported that both ILCs and TREM2 play important roles in pulmonary fibrosis, the relationships between TREM2 and ILCs and their exact roles in the pathogenesis of pulmonary fibrosis remain unclear. Research has shown that interleukin‐13 (IL‐13), a cytokine produced by ILCs, promotes the cleavage of TREM2 into soluble TREM2, which can prevent the apoptosis of macrophages and contribute to later chronic inflammatory disease [26]. We hypothesized that TREM2 influences BLM‐induced pulmonary inflammation and fibrosis by regulating ILCs. This study aimed to explore the role of TREM2 in regulating ILC activation in BLM‐induced pulmonary fibrosis to determine whether this pathway can serve as a target for the treatment of IPF.

## 2 Materials and Methods

### 2.1 Experimental Animals

Male C57BL/6 mice (8–12 weeks old, 20–25 g) were obtained from the National Experimental Animal Center (Taipei, Taiwan). They were maintained in pathogen‐free plastic cages with husk bedding at 25°C ± 2°C under a 12‐h light/dark cycle, with free access to food and water. TREM2 knockout mice on a C57BL/6 background, originally established in T.W. Mak's laboratory (Toronto, Canada), were maintained under specific pathogen‐free conditions at the National Yang Ming Chiao Tung University animal center. All animal procedures were approved by the Institutional Animal Care and Use Committee (IACUC #2016‐116).

#### 2.1.1 Animal Model

After anesthesia, mice received an intratracheal instillation of bleomycin (1.5 U/kg in 50 μL PBS; Merck, Darmstadt, Germany) to induce pulmonary fibrosis, following a modified protocol from a previous study [27]. Control mice received 50 μL PBS. Animals were sacrificed on Days 3, 7, and 14; control samples were collected at corresponding time points. Each group included three to five mice.

### 2.2 Histology and Immunohistochemistry (IHC)

Lung tissues were fixed in 4% paraformaldehyde for 10 min, paraffin‐embedded, and sectioned at 4 μm. Sections were stained for collagen‐1 (1:100, ab34710, Abcam), GATA‐3 (1:100, ab199428, Abcam), RORC (1:100, 13205‐1‐AP, Proteintech), TGF‐β (1:100, ARG10002, Arigo Biolaboratories), and α‐SMA (1:100, 14395‐1‐AP, Proteintech) using the EnVision + Dual Link System‐HRP (DAB+) kit (K4065, DAKO). After deparaffinization, rehydration, and antigen retrieval in 0.01 M citrate buffer (pH 6.0), endogenous peroxidase was quenched with 3% H_2_O_2_ for 10 min. Sections were blocked with kit buffer, incubated with secondary polymer‐HRP complexes for 30 min, and visualized using DAB substrate. Counterstaining was performed with hematoxylin (109249, Merck) for 10 s, followed by rinsing in running water for 10 min. Images were captured with an Olympus AX80 microscope, and the percentage of IHC‐positive area was quantified using Image‐Pro Plus software (Media Cybernetics, USA). To evaluate the severity of acute lung injury, a standardized histological lung injury scoring system was applied, as detailed in Data S1.

### 2.3 Masson's Trichrome Staining

The lung tissue samples were first fixed in 4% paraformaldehyde for 10 min and then embedded in paraffin. The samples were subsequently sectioned into slices measuring 4 μm in thickness. For staining, a Trichrome Stain Kit (#ab150686, Abcam, Cambridge, UK) was used, and we strictly adhered to the guidelines provided by the manufacturer. Pulmonary fibrosis severity was assessed using the Ashcroft scale, as detailed in Data S1.

### 2.4 Enzyme‐Linked Immunosorbent Assay

Lung tissue was homogenized in lysis buffer composed of radioimmunoprecipitation assay (RIPA) buffer (475 μL), a reagent cocktail (5 μL), and 0.1 M Na3VO4 (20 μL). This mixture was then centrifuged at 20,000 rpm for 10 min at 4°C. After centrifugation, the mixture was stored at −20°C until further procedures were performed. Tissue‐protective amphiregulin (AREG) levels in lung tissue were analyzed using Quantikine ELISA kits (M6000B, R&D Systems Inc., Minneapolis, MN, USA).

### 2.5 Western Blotting

Mouse lung tissues were homogenized in lysis buffer containing protease inhibitors (1862209, Thermo, Waltham, MA, USA). Equal protein amounts were separated by 7.5%–10% SDS‐PAGE and transferred to PVDF membranes. Blots were blocked with 5% milk in TBST, then probed with primary antibodies against collagen‐1 (1:1000, ab34710, Abcam, Cambridge, UK), TGF‐β (1:500, #3711, Cell Signaling Technology, Danvers, MA, USA), α‐SMA (1:1500, 14395‐1‐AP, Proteintech, Rosemont, IL, USA), and β‐actin (1:5000, 20536‐1‐AP, Proteintech, Rosemont, IL, USA). After incubation with HRP‐conjugated secondary antibodies, signals were detected using enhanced chemiluminescence (Pierce, Thermo Fisher Scientific) and quantified by ImageJ (NIH, Bethesda, MD, USA). Protein levels were normalized to β‐actin.

### 2.6 Immunofluorescence

The tissue sections were deparaffinized in xylene, rehydrated in ethanol, and subjected to antigen retrieval in 0.01 M citrate buffer (pH 6.0). After blocking with 3% FBS in PBS for 1 h at room temperature, sections were incubated overnight at 4°C with GATA‐3 (ab199428, Abcam) and RORγt (ab78007, Abcam) antibodies. The next day, Alexa Fluor 488–conjugated (ab150077, Abcam) and Cy5–conjugated (ab6564, Abcam) secondary antibodies (1:400) were applied at 37°C for 2 h. Slides were mounted with DAPI‐containing medium (H‐1200, Vector Laboratories) and imaged using a FluoView confocal microscope (FV10i, Olympus).

### 2.7 Innate Lymphocyte Adoptive Transfer

Lung cells were isolated 3 days after BLM treatment. Whole lungs were minced in RPMI‐1640 (GE Healthcare HyClone) containing 1% pen/strep, 5% FBS, and 0.005% collagenase (Sigma). Following enzymatic digestion, single‐cell suspensions were prepared for ILC enrichment. Cell separation was performed using the MACS system with biotin anti‐mouse lineage panels (133307, BioLegend), CD45 microbeads (130‐097‐153), and streptavidin microbeads (130‐048‐102, Miltenyi Biotec). Specifically, lineage‐positive cells were first depleted using the biotin anti‐mouse Lineage Panel, which includes antibodies against TER‐119, CD11b, Ly‐6G/Ly‐6C, CD3e, and CD45R/B220, followed by magnetic removal with streptavidin MicroBeads. The remaining lineage‐negative cells were subsequently enriched by positive selection using CD45 MicroBeads to obtain a Lin^−^CD45^+^ ILC‐enriched population. Each mouse received 1 × 10^4^ innate lymphocytes (pooled from three donors) via tail vein injection. Lungs were harvested on Days 3, 7, and 14, and ILCs were counted using a hemocytometer.

### 2.8 Flow Cytometry

Isolated ILCs were washed and resuspended in staining buffer. For intracellular subset characterization, cells were fixed, permeabilized, and stained with an anti‐GATA3 antibody according to the manufacturer's instructions. Data were acquired on a BD Biosciences flow cytometer and analyzed using the associated software. Lymphocytes were gated based on forward and side scatter (FSC/SSC) properties, and ILCs were identified within this gate. The proportion of GATA3^+^ cells was quantified using unstained samples to define negative gating thresholds.

### 2.9 Statistical Analysis

The data from these analyses are reported as either the mean ± standard deviation of the mean or standard deviation for each experimental group. Statistical evaluations were conducted using a two‐sample *t*‐test. A *p*‐value of less than 0.05 was considered statistically significant. Statistical analyses and histogram generation were performed using GraphPad Prism 6.0.

## 3 Results

### 3.1 TREM2 KO Mice Exhibit Increased Bleomycin‐Induced Lung Injury and Fibrosis

An in vivo BLM‐induced pulmonary fibrosis model that recapitulates key pathological features of IPF has been validated in a previous study [28]. Temporal profiling revealed lung inflammation by Day 3 after BLM administration, followed by prominent fibrosis at Day 14, culminating in maximal fibrosis with near‐complete parenchymal obliteration at Day 21 (Figure S1). Accordingly, we selected Day 14 post‐BLM exposure as the time point for assessing pulmonary inflammation and fibrosis severity.

**FIGURE 1:**
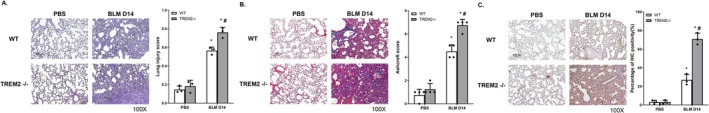
Triggering receptor expressed on myeloid cells‐2 knockout (TREM2 KO) promotes histopathological changes and exacerbates pulmonary inflammation and fibrosis in mice with bleomycin (BLM)‐induced pulmonary fibrosis. (A) HE staining. Hematoxylin and eosin (HE) staining and lung injury assessment revealed greater lung inflammation, fibrosis, and epithelial hyperplasia in the alveolar regions of the TREM2‐KO mice than in those of the wild‐type (WT) mice at 14 days post‐BLM injection. (B) Masson's trichrome staining. Mice with TREM2 KO and pulmonary fibrosis presented significantly more fibrosis than those in the BLM‐only group did, as determined by Masson's trichrome staining and the Ashcroft score. (C) IHC for collagen‐1. Immunohistochemical (IHC) analysis revealed a marked increase in collagen‐1 levels 14 days after BLM treatment, with TREM2 KO further increasing collagen‐1 levels after BLM injection. *p < 0.05 compared with the PBS group; #*p* < 0.05 compared with the BLM WT group. The *p*‐value was calculated using pairwise Student's *t*‐tests. PBS, phosphate‐buffered saline; WT, wild type; TREM2−/−, TREM2 knockout. *n* = 3–5 per group.

The lung inflammation and fibrosis triggered by intratracheal BLM administration were marked by increased inflammatory cell infiltration, widespread alveolar fibrosis, localized dense fibrosis, and alveolar duct epithelial hyperplasia, as shown by hematoxylin and eosin (HE) staining (Figure 1A). Our study focused on the role of TREM2 in BLM‐induced lung fibrosis by comparing lung sections from TREM2‐KO mice and wild‐type mice. Fourteen days post‐BLM exposure, the TREM2 KO mice presented significantly increased lung inflammation and fibrosis, as indicated by histological assessment and elevated lung injury scores (Figure 1A). Masson's trichrome staining and the Ashcroft score were used to assess the lung tissues, which further confirmed the development of fibrosis in these mice (Figure 1B). Additionally, TREM2 KO increased BLM‐induced fibrosis, as indicated by Masson's trichrome staining and the Ashcroft score (Figure 1B). IHC was performed to identify changes in proteins in the lung tissue. IHC of lung tissue revealed increased collagen‐1 levels 14 days after BLM treatment, with a notable increase in the TREM2‐KO mice, further confirming that TREM2 KO exacerbated BLM‐induced fibrosis (Figure 1C).

### 3.2 TREM2‐KO Mice Exhibit an Increase in ILC Expression

**FIGURE 2:**
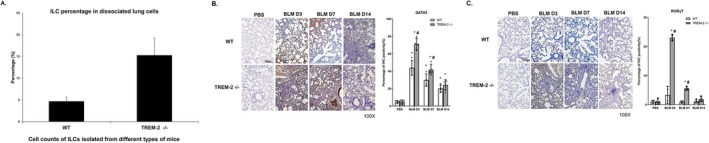
Triggering receptor expressed on myeloid cells‐2 knockout (TREM2 KO) increased innate lymphoid cell (ILC) recruitment in mice with pulmonary fibrosis. (A) ILC count on BLM treatment Day 3 in WT and TREM2−/− mice. Increased accumulation of ILCs in the lungs of mice with bleomycin (BLM)‐induced pulmonary fibrosis compared with the control mice, with the TREM2**‐**KO mice exhibiting a notably increased proportion of ILCs. (B) IHC for GATA3. Immunohistochemistry (IHC) showed that bleomycin increased GATA3 expression, which was further enhanced in TREM2‐KO mice and peaked on Day 3 post‐BLM. (C) IHC for RORγt. RORγt immunostaining exhibited a similar pattern, with higher levels in TREM2‐KO mice and a maximal signal on Day 3 after BLM exposure. *p < 0.05 compared with the PBS group; #*p* < 0.05 compared with the BLM WT group. The *p*‐value was calculated using pairwise Student's *t*‐tests. PBS, Phosphate‐buffered saline; WT, Wild type; TREM2−/−, TREM2 KO. *n* = 3–5 per group.

In mice with BLM‐induced pulmonary fibrosis, there was a notable accumulation of ILCs in the lungs, with the TREM2‐KO mice showing a significant increase in the ILC proportion (Figure 2A). IHC analysis revealed increased expression of GATA3, which is essential for ILC2 development and maintenance (Figure 2B), as well as RORγt, a known progenitor marker for ILC3s (Figure 2C) [29, 30]. These expressions rose at 3, 7, and 14 days after BLM injection. Both signals were markedly stronger in TREM2‐KO mice (Figure 2B,C), peaking on Day 3 and declining thereafter. Consequently, we isolated ILCs for adoptive transfer from mice on post‐BLM Day 3.

### 3.3 Increased BLM‐Induced Lung Injury and Fibrosis in Mice With Adoptive Transfer of ILCs

**FIGURE 3:**
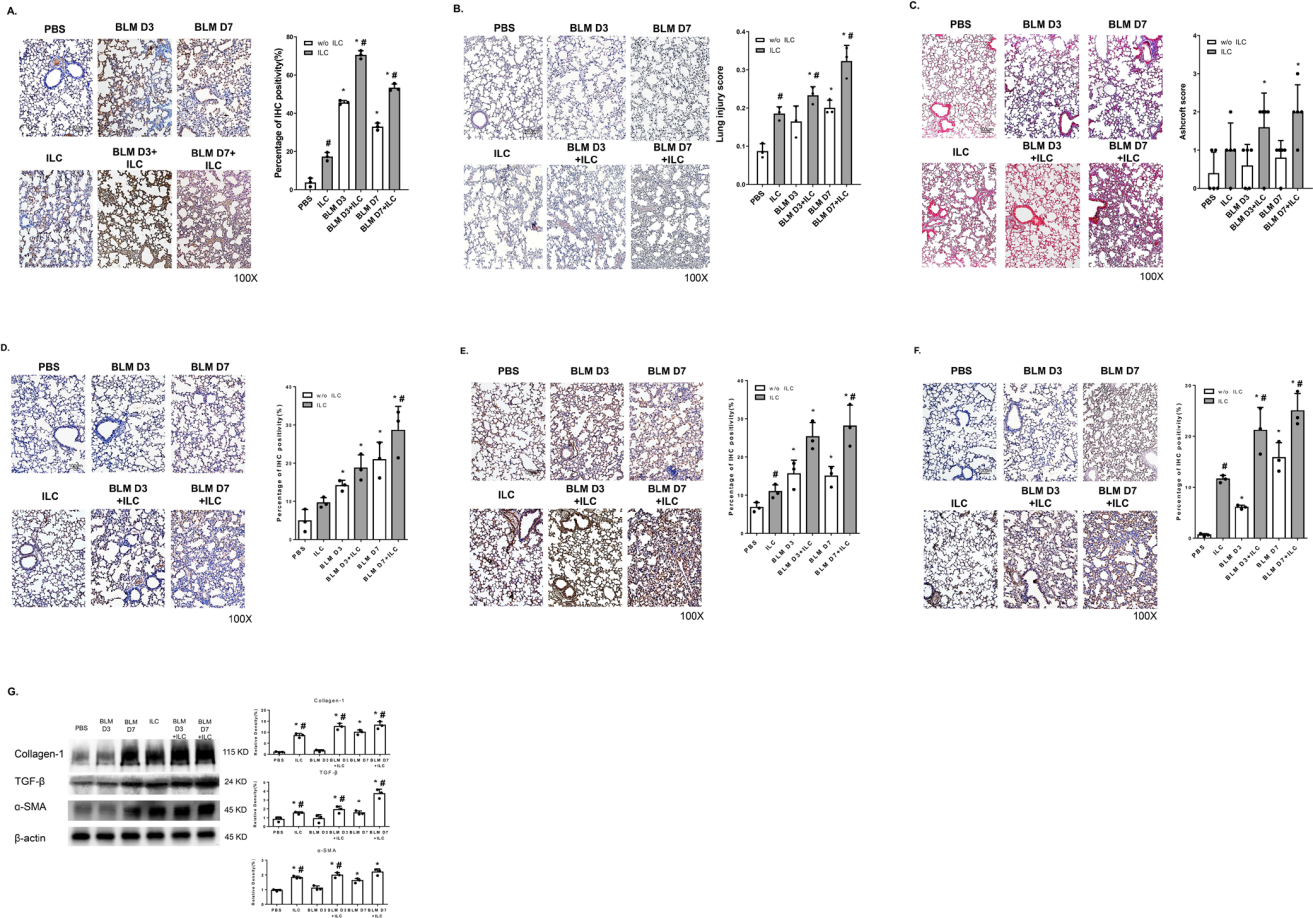
Increased bleomycin (BLM)‐induced lung injury and fibrosis in mice with adoptive transfer of innate lymphoid cells (ILCs). ILCs (1 × 10^4^ cells/mouse) isolated from mice 3 days after intratracheal injection with BLM were intravenously administered to healthy mice via the tail vein. (A) IHC for GATA 3 in ILCs used for in vivo adoptive transfer. Immunohistochemical (IHC) staining revealed that GATA 3 levels were significantly greater at both 3 and 7 days post‐BLM injection, especially in the mice that received adoptive transfer of ILCs before BLM injection and at 3 and 7 days post‐injection. (B) HE staining of ILCs used for in vivo adoptive transfer. Hematoxylin and eosin (HE) staining, along with lung injury scores, indicated increased lung inflammation in the mice at both 3 and 7 days following the adoptive transfer of ILCs after BLM injection. (C) Masson's trichrome staining of ILCs used for in vivo adoptive transfer. Mice that received adoptive ILC transfer exhibited more severe pulmonary fibrosis, as evidenced by Masson's trichrome staining and the Ashcroft score. (D) IHC for TGF‐β in ILCs used for in vivo adoptive transfer. IHC staining revealed that TGF‐β levels were significantly elevated at both 3 and 7 days post‐BLM injection. The adoptive transfer of ILCs further increased TGF‐β levels at 7 days after BLM injection. (E) IHC for α‐SMA in ILCs used for in vivo adoptive transfer. IHC staining revealed that α‐SMA levels were significantly increased at both 3 and 7 days post‐BLM injection, with a further increase following the adoptive transfer of ILCs before BLM injection and at 7 days post‐injection. (F) IHC for collagen‐1 in ILCs used for in vivo adoptive transfer. IHC staining revealed a significant increase in collagen‐1 levels at both 3 and 7 days post‐BLM injection. The adoptive transfer of ILCs further increased collagen‐1 levels before BLM injection and at 3 and 7 days post‐injection. (G) Whole‐lung WB analysis of ILCs used for in vivo adoptive transfer. Western blot and semiquantitative analyses were used to assess protein expression in the lung homogenates of the mice treated with BLM, both with and without the adoptive transfer of ILCs. The analysis, which was conducted with specific antibodies, revealed that the protein levels of collagen‐1, TGF‐β, and α‐SMA were significantly elevated in the mice 7 days after BLM injection. Notably, the adoptive transfer of ILCs further increased the levels of collagen‐1, TGF‐β, and α‐SMA prior to BLM injection and at 3 and 7 days post‐injection. *p < 0.05 compared with the PBS group; #*p* < 0.05 compared with the BLM without ILC group. The *p*‐value was calculated using pairwise Student's *t*‐tests. PBS, Phosphate‐buffered saline; ILC group, Mice with the adoptive transfer of innate lymphoid cells. *n* = 3–5 per group.

To characterize the composition of ILCs used in experiments, cells were analyzed after MACS isolation. Flow cytometric analysis showed that approximately 82% of the isolated cells were ILCs. Within this population, GATA3^+^ cells comprised 55.7% (Figure S2). Lung inflammation and fibrosis were observed in mice following the in vivo adoptive transfer of the ILC‐enriched population, which were extracted from mice 3 days after intratracheal injection with BLM. IHC revealed increased expression of GATA 3 at both 3 and 7 days post‐BLM injection, with significantly increased expression in the mice that received the adoptive transfer of the ILC‐enriched population (Figure 3A). Significant increases in lung inflammation and fibrosis were noted in the mice following the adoptive transfer of the ILC‐enriched population. This result was demonstrated by HE staining and increased lung injury scores (Figure 3B). Additionally, compared with the mice that did not receive the adoptive transfer of the ILC‐enriched population, those that received the adoptive transfer presented increased fibrosis, as demonstrated by Masson's trichrome staining of lung tissue and Ashcroft scoring (Figure 3C). The increased expression of TGF‐β (Figure 3D), α‐SMA (Figure 3E), and collagen‐1 (Figure 3F) in the mice with the adoptive transfer of the ILC‐enriched population further substantiated these findings, as demonstrated by IHC staining. Protein expression levels in murine lung homogenates were analyzed using Western blotting, and the results are presented in Figure 3G. The protein levels of collagen‐1, TGF‐β, and α‐SMA were elevated in the mice that underwent the adoptive transfer of the ILC‐enriched population.

### 3.4 Increased ILCs Expression in Mice That Received Adoptive Transfer of ILC‐Enriched Population Isolated From TREM2‐KO Mice

**FIGURE 4:**
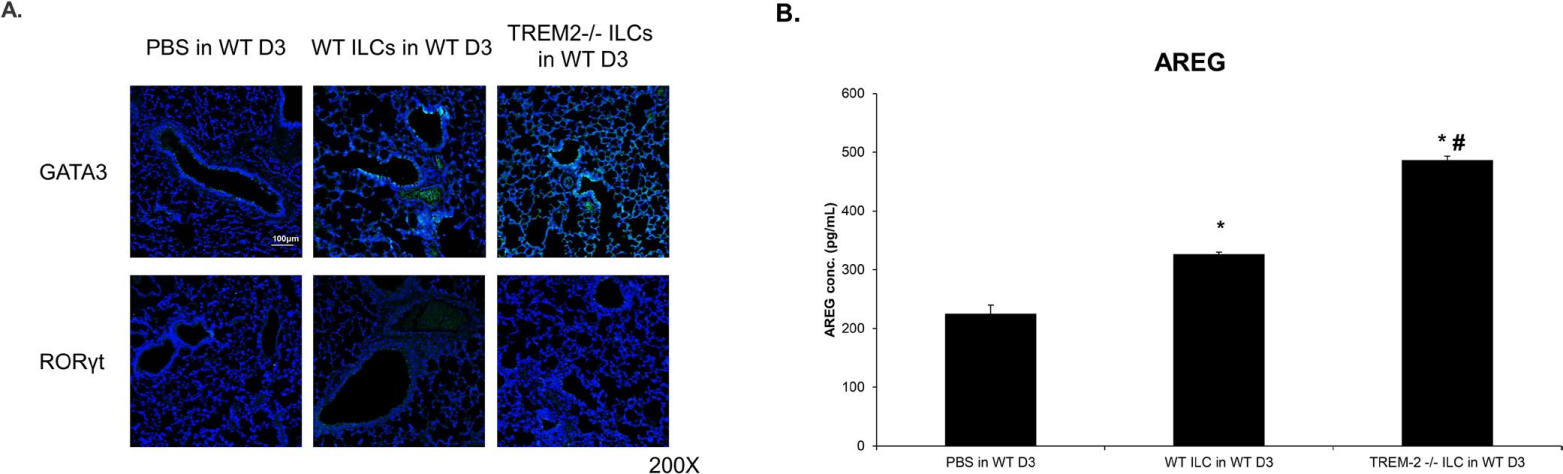
Increased ILC2 expression in TREM2 KO mice. (A) IF staining of lung samples. Immunofluorescence (IF) staining of lung tissues revealed an increase in GATA 3 expression in the mice that had undergone adoptive transfer of ILCs. This elevation in GATA 3 levels was notably more pronounced in the mice that received ILCs derived from TREM2 knockout (KO) mice than in those that received ILCs from wild‐type (WT) mice. In contrast, no RORγt expression was observed in the mice, regardless of whether they received ILCs from WT or TREM2‐KO mice. (B) Whole‐lung ELISA for AREG. An enzyme‐linked immunosorbent assay (ELISA) conducted on whole‐lung extracts revealed a significant increase in tissue‐protective amphiregulin (AREG) levels in the mice that underwent adoptive transfer of innate lymphoid cells (ILCs). Notably, AREG levels were greater in the mice that received ILCs derived from TREM2 knockout (KO) mice than in those that received ILCs extracted from wild‐type (WT) mice, particularly at 3 days after receiving ILCs. *p < 0.05 compared with the control group; #*p* < 0.05 compared with the group receiving WT ILCs. The *p*‐value was calculated using pairwise Student's *t*‐tests. PBS, Phosphate‐buffered saline; WT, Wild type; TREM2−/−, TREM2 knockout. *n* = 3–5 per group.

ILC‐enriched population isolated from TREM2‐KO and WT mice 3 days after intratracheal injection of BLM were intravenously administered to healthy mice. In Figure 4A, IF staining of lung tissues revealed an increase in GATA 3 expression in mice that had undergone adoptive transfer of ILC‐enriched population. Additionally, this increase in GATA 3 levels was more pronounced in the mice that received ILC‐enriched population derived from TREM2‐KO mice than in those that received ILC‐enriched population extracted from WT mice. However, this increase was not observed in RORγt expression, a critical factor in the development of ILC3s [31]. ELISAs of whole‐lung extracts revealed that the level of AREG, a ligand for the epidermal growth factor receptor known to be secreted by ILC2s and play a crucial role in fibroblast production [32], was elevated in the mice that received adoptive transfer of ILC‐enriched population. Furthermore, AREG levels were greater in the mice that received adoptive transfer of ILC‐enriched population derived from TREM2‐KO mice than in those that received ILC‐enriched population extracted from WT mice (Figure 4B).

### 3.5 Mice That Received Adoptive Transfer of ILCs From TREM‐KO Mice Exhibited Increased Lung Injury and Fibrosis

**FIGURE 5:**
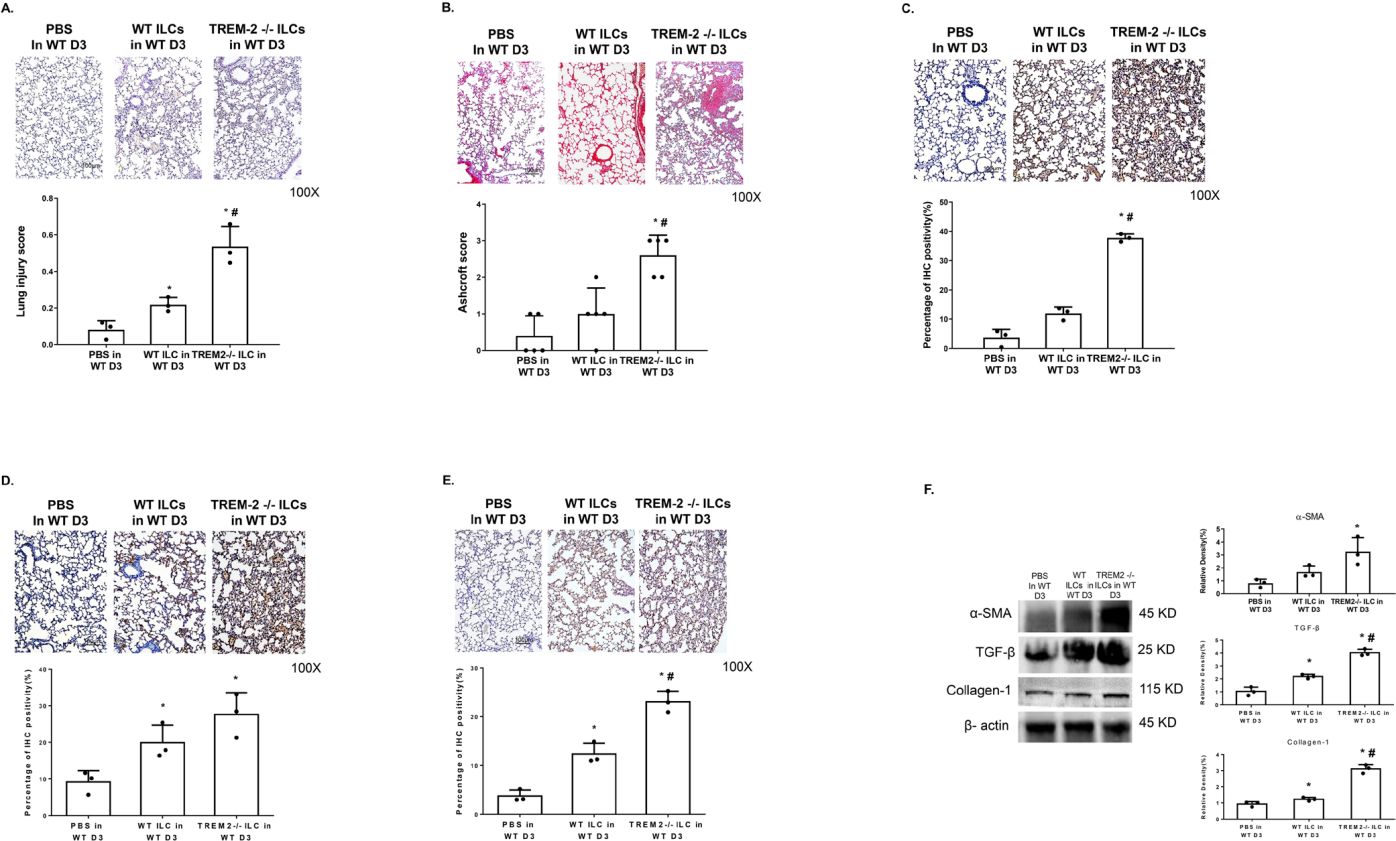
Mice that received adoptive transfer of innate lymphoid cells (ILCs) from TREM2 knockout (KO) mice presented increased lung injury and fibrosis. (A) HE staining after adoptive transfers of ILCs from WT and TREM2‐KO mice. There was a significant increase in lung inflammation in the mice receiving ILCs, with notably greater severity in those that received ILCs from TREM2‐KO mice, especially at 3 days after transfer, than in those that received ILCs from wild‐type (WT) mice. (B) Masson staining after adoptive transfers of ILCs from WT and TREM2‐KO mice. Masson's trichrome staining and the Ashcroft score revealed more advanced pulmonary fibrosis in the mice with adoptive transfer of ILCs. The extent of lung fibrosis was significantly more severe in the mice that received ILCs from TREM2‐KO mice than in those from WT mice, particularly at 3 days post‐transfer. (C) Lung IHC analysis for TGF‐β after adoptive transfers of ILCs from WT and TREM2‐KO mice. Immunohistochemistry (IHC) staining demonstrated that TGF‐β levels were significantly elevated 3 days after receiving adoptive transfer of ILCs from TREM2‐KO mice. (D) IHC analysis of α‐SMA in lungs after adoptive transfers of ILCs from WT and TREM2‐KO mice. IHC staining also revealed an increase in α‐SMA levels at 3 days following the adoptive transfer of ILCs. (E) Lung IHC analysis for collagen‐1 after adoptive transfers of ILCs from WT and TREM2‐KO mice. IHC staining revealed a significant increase in collagen‐1 levels at 3 days following the adoptive transfer of ILCs, with an even greater increase observed in those derived from TREM2‐KO mice than in those derived from WT mice. (F) Whole‐lung WB analysis after adoptive transfers of ILCs from WT and TREM2‐KO mice. Western blot and semiquantitative analyses were used to measure protein expression in lung homogenates from mice, which revealed that collagen‐1, TGF‐β, and α‐SMA levels were greater on Day 3 following adoptive ILC transfer. Proteins were further elevated in recipients of ILCs from TREM2‐KO mice compared with those from WT mice. *p < 0.05 compared with the PBS group; #*p* < 0.05 compared with the group receiving WT ILCs. The *p*‐value was calculated using pairwise Student's *t*‐tests. PBS, Phosphate‐buffered saline; WT, Wild type; TREM2−/−, TREM2 knockout. *n* = 3–5 per group.

Lung inflammation was markedly more severe in the mice that received ILC‐enriched population derived from TREM2‐KO mice than in those that received ILC‐enriched population from wild‐type WT mice. This finding was confirmed by both HE staining and lung injury scoring (Figure 5A). Additionally, Masson's trichrome staining and the Ashcroft score indicated more advanced pulmonary fibrosis in the mice with ILC‐enriched population from TREM2 KO mice (Figure 5B). Compared with the mice that received ILC‐enriched population from WT mice, those with ILCs from TREM2‐KO mice presented greater expression of TGF‐β (Figure 5C), α‐SMA (Figure 5D), and collagen‐1 (Figure 5E), as shown by IHC staining. Western blot analysis further revealed that the protein levels of collagen‐1, TGF‐β, and α‐SMA were elevated in the mice with adoptive transfer of ILC‐enriched population, with a more pronounced increase in the mice that received ILC‐enriched population from TREM2‐KO mice than in those that received ILC‐enriched population from WT mice (Figure 5F).

## 4 Discussion

In our current study, we initially focused on a pulmonary fibrosis model in mice to evaluate the role of TREM2. TREM2 KO mice exhibited increased lung injury and fibrosis following intratracheal BLM injection. We subsequently reported that mice that underwent adoptive transfer of ILC‐enriched population experienced increased lung injury and fibrosis. Finally, there was an increase in ILC2 expression in TREM2‐KO mice, and the mice that received adoptive transfer of ILC‐enriched population from TREM2‐KO mice presented the most severe lung injury and fibrosis.

The increase in pulmonary fibrosis in TREM2‐KO mice suggests that TREM2 plays a protective role in lung fibrosis. In models of fibrosis‐associated hepatoma, TREM2 has been shown to have protective effects by diminishing fibrosis and liver damage [33]. Similarly, in research focused on nonalcoholic steatohepatitis, a lack of hematopoietic TREM2 resulted in worsened hepatitis, increased cell death, and increased fibrosis [34]. These earlier studies corroborate our findings on the antifibrotic effects of TREM2.

The subsequent question is how TREM2 inhibits pulmonary fibrosis. Our study revealed that TREM2‐KO mice presented elevated ILC2 expression. Mice that underwent adoptive transfer of ILC‐enriched population experienced increased lung injury and fibrosis after BLM induction. Notably, the mice that received ILC‐enriched population from TREM2‐KO donors presented more severe lung injury and fibrosis than did those that received ILC‐enriched population from WT mice. These findings suggest that TREM2 may protect against lung fibrosis by regulating ILC‐enriched population. Of note, several studies found blockade of TREM2 might also reduce the severity of pulmonary fibrosis [20, 24, 25]. On the other hand, our study found the protective role of TREM2 in lung inflammation and subsequent fibrosis, via regulation of ILC‐enriched population.

However, our findings contrast with some studies suggesting TREM2 promotes pulmonary fibrosis [19, 20, 24, 25]. This discrepancy is most likely attributable to cell‐type–specific functions of TREM2. Whereas macrophage‐centered studies implicate TREM2 in fibrosis through lipid‐metabolic pathways [24, 25], our data identify TREM2 as a protective regulator that restrains pro‐fibrotic ILC activity. These observations underscore the context‐dependent, multi‐faceted nature of TREM2, suggesting that its net effect on fibrosis is dictated by the dominant cellular pathway engaged. Definitive clarification will require future studies employing cell‐specific genetic models.

Chronic post viral disease is characterized by an increase in M2‐differentiated pulmonary macrophages, necessitating TREM2 [26]. The activation of ILC2s leads to the production of several Th2 cytokines, including IL‐13 [35]. As IL‐13 levels increase, virus replication leads to increased macrophage and TREM2 levels in lung tissues, which hinders macrophage apoptosis in acute diseases. After infection is resolved, IL‐13 promotes the conversion of TREM2 into its soluble variant, inhibiting macrophage apoptosis [26]. These outcomes might explain the communication among ILCs, TREM2, and M2 macrophages during acute infections, resulting in chronic inflammatory diseases [36]. For ILCs, numerous studies have confirmed that ILCs induce lung fibrosis [8, 9, 10, 11]. Therefore, in cases of pulmonary fibrosis, TREM2 may also influence the fibrotic condition by regulating ILCs, which aligns with the findings of our study.

TREM2 activation has therapeutic potential for several diseases [16]. In neurodegenerative disorders, in vitro studies have shown that overexpressing TREM2 reduces inflammation and increases microglial phagocytosis [37]. Increasing TREM2 expression through lentivirus transfection ameliorates cognitive impairment and neuropathological alterations in mice with Alzheimer's disease [38]. AL002, a novel investigational humanized monoclonal antibody that binds to TREM2, was tested in a Phase II study in patients with Alzheimer's disease; this trial shed light on the role of TREM2 in neuroprotection [39]. The above therapy, which promotes TREM2, may also hold therapeutic potential for pulmonary fibrosis based on our findings.

Our study had several limitations. First, the pathogenesis of pulmonary fibrosis involves various types of inflammatory cells with different immune pathways. While we focused on the TREM2‐ILC axis, TREM2 is predominantly expressed on macrophages, and ILC shifts may be partially influenced by indirect downstream effects from these cells during initial priming. Although our adoptive transfer data confirm that TREM2‐KO ILC‐enriched population drives fibrosis, the exact macrophage‐ILC crosstalk remains a target for future research using a cell‐specific knockout model. Second, we did not evaluate the direct treatment effect of TREM2, such as the use of supplemental agents to increase the level of TREM2 or the use of TREM2‐overexpressing mice. While our data establish TREM2 as a critical protective regulator, further research is required to determine whether boosting TREM2 levels can effectively halt or reverse already formed fibrotic scarring. Third, single‐marker IHC for GATA‐3 and RORγt has inherent limitations in specifically identifying ILCs, as these transcription factors are also expressed by adaptive CD4^+^ T helper cells, including Th2 and Th17 subsets. However, we employed a lineage‐negative (Lin^−^) depletion strategy in our adoptive transfer experiments to enrich for the innate compartment. Therefore, our findings regarding ILC involvement are primarily based on functional adoptive transfer experiments using Lin^−^ cells, rather than on transcription factor expression in tissue alone. Forth, a limitation of our study is the lack of direct evidence, such as cell‐tracking visualization, for the pulmonary homing of intravenously transferred ILC‐enriched population. However, circulating ILC2s are established to home to inflamed lungs and drive tissue remodeling [40]. Consistently, our finding that transferred ILCs significantly increased pro‐fibrotic markers supports their recruitment and functional activity within the pulmonary environment. Fifth, although we examined several markers representing ILC2s and ILC3s (AREG, GATA3, and RORγt), we lacked the ability to separate ILC2s specifically, leading to the adoptive transfer of the whole ILC‐enriched population. Due to this issue, it is unclear which type of ILC is primarily responsible for pulmonary fibrosis. Given that GATA3 was the predominant marker observed in the TREM2–ILC–pulmonary fibrosis axis, it is plausible that ILC2s represent the major ILC subtype involved. However, this hypothesis requires confirmation through further experiments. Finally, our study lacks direct in vitro mechanistic models, which limits the causal interpretation of the TREM2–ILC axis. Therefore, these findings should be considered functional and hypothesis‐generating, providing a valuable foundation for future research to definitively dissect the underlying molecular mechanisms. However, our study also has several strengths. First, to our knowledge, this is the first fundamental study to investigate the role of TREM2 in pulmonary fibrosis through the regulation of ILC‐enriched population. Second, the methodology of adoptive transfer of ILC‐enriched population from BLM‐injected WT/TREM2‐KO mice clarified the TREM2‐ILC‐pulmonary fibrosis pathway. Our data suggest that TREM2 KO can promote pulmonary fibrosis by increasing the ILC‐enriched population. Thus, treatments that activate TREM2 may alleviate lung fibrosis. However, further research is needed to fully elucidate the positive effects of TREM2 in fibrotic lung diseases.

In conclusion, TREM2‐KO exacerbated lung injury and fibrosis after BLM exposure, accompanied by elevated ILC levels. Adoptive transfer of ILC‐enriched population from TREM2‐KO mice similarly intensified fibrosis. Overall, activating TREM2 appears to suppress ILC activity and mitigate BLM‐induced pulmonary fibrosis.

## Funding

This work was supported by Taipei Veterans General Hospital (V114B‐017, V114EA‐008, V115EA‐007, V112B‐031, V113B‐015, V114B‐009, V112C‐068, V113C‐007, V112D65‐003‐MY2‐1, V112D65‐003‐MY2‐2, V115B‐004). The National Science and Technology Council, Taiwan (NSTC 114‐2314‐B‐075‐009, NSTC 112‐2314‐B‐075‐050, 113‐2314‐B‐075‐045, NSTC 112‐2314‐B‐A49‐040). Cancer and Immunology Research Center of National Yang Ming Chiao Tung University (NYCU) (112 W 31101, 113 W 031101, 114 W 031101).

## Ethics Statement

All the experiments were conducted in accordance with the Institutional Animal Care and Use Committee‐approved protocols (TVGH IACUC No. 2016‐116).

## Conflicts of Interest

The authors declare no conflicts of interest.

## Supporting information


**Data S1:** kjm270209‐sup‐0001‐Figures.pdf.
**Figure S1:** HE and Masson staining of mouse lung tissue following bleomycin treatment. In C57BL/6 mice, a single intratracheal (i.t.) administration of bleomycin (BLM) led to a progressive course of lung injury and fibrosis. Histopathological assessment showed evident inflammatory damage as early as Day 3, prominent interstitial fibrosis with collagen accumulation and tissue architecture disruption by Day 14, and almost total loss of normal parenchymal structure by Day 21. *p < 0.05 compared with the control group. The *p*‐value was calculated using pairwise Student's *t*‐tests. *n* = 3–5 per group.
**Figure S2:** Flow cytometric characterization of MACS‐isolated ILC‐enriched population. Cells were analyzed by flow cytometry after MACS isolation to verify ILC purity and subset composition. Lymphocytes were gated by FSC/SSC, showing that ~82% of events fell within the lymphocyte gate, confirming successful enrichment. Intracellular GATA3 staining identified ILC2s within this population, with GATA3^+^ cells accounting for 55.7% of isolated ILC‐enriched population.

## Data Availability

The data that support the findings of this study are available from the corresponding author upon reasonable request.

## References

[1] D. J. Lederer and F. J. Martinez , “Idiopathic Pulmonary Fibrosis,” New England Journal of Medicine 378, no. 19 (2018): 1811–1823. 29742380 10.1056/NEJMra1705751

[2] A. L. Olson , A. H. Gifford , N. Inase , E. R. F. Pérez , and T. Suda , “The Epidemiology of Idiopathic Pulmonary Fibrosis and Interstitial Lung Diseases at Risk of a Progressive‐Fibrosing Phenotype,” European Respiratory Review 27, no. 150 (2018): 180077. 30578336 10.1183/16000617.0077-2018 PMC9489016

[3] Y. H. Khor , Y. Ng , H. Barnes , N. S. L. Goh , C. F. McDonald , and A. E. Holland , “Prognosis of Idiopathic Pulmonary Fibrosis Without Anti‐Fibrotic Therapy: A Systematic Review,” European Respiratory Review 29, no. 157 (2020): 190158. 32759374 10.1183/16000617.0158-2019 PMC9488716

[4] E. Vivier , D. Artis , M. Colonna , et al., “Innate Lymphoid Cells: 10 Years on,” Cell 174, no. 5 (2018): 1054–1066. 30142344 10.1016/j.cell.2018.07.017

[5] H. Morita , K. Arae , H. Unno , et al., “An Interleukin‐33‐Mast Cell‐Interleukin‐2 Axis Suppresses Papain‐Induced Allergic Inflammation by Promoting Regulatory T Cell Numbers,” Immunity 43, no. 1 (2015): 175–186. 26200013 10.1016/j.immuni.2015.06.021 PMC4533925

[6] M. Ebbo , A. Crinier , F. Vély , and E. Vivier , “Innate Lymphoid Cells: Major Players in Inflammatory Diseases,” Nature Reviews Immunology 17, no. 11 (2017): 665–678. 10.1038/nri.2017.86 28804130

[7] U. I. Can , S. E. Stenske , M. D. Rosenbaum , and R. L. Reinhardt , “Rapid Group‐2 Innate Lymphoid Cell Mobilization From the Intestine Aids in Early Lung Defense and Repair,” Cell Reports 44, no. 7 (2025): 115868. 40543040 10.1016/j.celrep.2025.115868 PMC12352581

[8] D. Li , R. Guabiraba , A. G. Besnard , et al., “IL‐33 Promotes ST2‐Dependent Lung Fibrosis by the Induction of Alternatively Activated Macrophages and Innate Lymphoid Cells in Mice,” Journal of Allergy and Clinical Immunology 134, no. 6 (2014): 1422–32.e11. 24985397 10.1016/j.jaci.2014.05.011 PMC4258609

[9] N. Otaki , Y. Motomura , T. Terooatea , et al., “Activation of ILC2s Through Constitutive IFNγ Signaling Reduction Leads to Spontaneous Pulmonary Fibrosis,” Nature Communications 14, no. 1 (2023): 8120. 10.1038/s41467-023-43336-6 PMC10721793 38097562

[10] E. Hams , M. E. Armstrong , J. L. Barlow , et al., “IL‐25 and Type 2 Innate Lymphoid Cells Induce Pulmonary Fibrosis,” Proceedings of the National Academy of Sciences of the United States of America 111, no. 1 (2014): 367–372. 24344271 10.1073/pnas.1315854111 PMC3890791

[11] T. Wohlfahrt , S. Usherenko , M. Englbrecht , et al., “Type 2 Innate Lymphoid Cell Counts Are Increased in Patients With Systemic Sclerosis and Correlate With the Extent of Fibrosis,” Annals of the Rheumatic Diseases 75, no. 3 (2016): 623–626. 26338035 10.1136/annrheumdis-2015-207388

[12] S. Shirley , H. Ichise , V. Di Natale , et al., “A Vasculature‐Resident Innate Lymphoid Cell Population in Mouse Lungs,” Nature Communications 16, no. 1 (2025): 3718. 10.1038/s41467-025-58982-1 PMC12009297 40253407

[13] J. M. Felton , R. Duffin , C. T. Robb , et al., “Facilitation of IL‐22 Production From Innate Lymphoid Cells by Prostaglandin E(2) Prevents Experimental Lung Neutrophilic Inflammation,” Thorax 73, no. 11 (2018): 1081–1084. 29574419 10.1136/thoraxjnl-2017-211097 PMC6200127

[14] A. Ardain , J. Z. Porterfield , H. N. Kløverpris , and A. Leslie , “Type 3 ILCs in Lung Disease,” Frontiers in Immunology 10 (2019): 92. 30761149 10.3389/fimmu.2019.00092 PMC6361816

[15] M. S. Wilson , S. K. Madala , T. R. Ramalingam , et al., “Bleomycin and IL‐1β–Mediated Pulmonary Fibrosis Is IL‐17A Dependent,” Journal of Experimental Medicine 207, no. 3 (2010): 535–552. 20176803 10.1084/jem.20092121 PMC2839145

[16] A. Deczkowska , A. Weiner , and I. Amit , “The Physiology, Pathology, and Potential Therapeutic Applications of the TREM2 Signaling Pathway,” Cell 181, no. 6 (2020): 1207–1217. 32531244 10.1016/j.cell.2020.05.003

[17] J. A. Hamerman , J. R. Jarjoura , M. B. Humphrey , M. C. Nakamura , W. E. Seaman , and L. L. Lanier , “Cutting Edge: Inhibition of TLR and FcR Responses in Macrophages by Triggering Receptor Expressed on Myeloid Cells (TREM)‐2 and DAP12,” Journal of Immunology 177, no. 4 (2006): 2051–2055. 10.4049/jimmunol.177.4.2051 16887962

[18] D. E. Byers , K. Wu , G. Dang‐Vu , et al., “Triggering Receptor Expressed on Myeloid Cells‐2 Expression Tracks With M2‐Like Macrophage Activity and Disease Severity in COPD,” Chest 153, no. 1 (2018): 77–86. 29017955 10.1016/j.chest.2017.09.044 PMC5812763

[19] D. Wendisch , O. Dietrich , T. Mari , et al., “SARS‐CoV‐2 Infection Triggers Profibrotic Macrophage Responses and Lung Fibrosis,” Cell 184, no. 26 (2021): 6243–61.e27. 34914922 10.1016/j.cell.2021.11.033 PMC8626230

[20] Q. Luo , D. Deng , Y. Li , et al., “TREM2 Insufficiency Protects Against Pulmonary Fibrosis by Inhibiting M2 Macrophage Polarization,” International Immunopharmacology 118 (2023): 110070. 37003186 10.1016/j.intimp.2023.110070

[21] E. Iizasa , Y. Chuma , T. Uematsu , et al., “TREM2 Is a Receptor for Non‐Glycosylated Mycolic Acids of Mycobacteria That Limits Anti‐Mycobacterial Macrophage Activation,” Nature Communications 12, no. 1 (2021): 2299. 10.1038/s41467-021-22620-3 PMC8052348 33863908

[22] V. Y. Su , K. Y. Yang , S. H. Chiou , et al., “Induced Pluripotent Stem Cells Regulate Triggering Receptor Expressed on Myeloid Cell‐1 Expression and the p38 Mitogen‐Activated Protein Kinase Pathway in Endotoxin‐Induced Acute Lung Injury,” Stem Cells 37, no. 5 (2019): 631–639. 30681755 10.1002/stem.2980

[23] H. Liu , L. Zhang , Z. Liu , et al., “Galectin‐3 as TREM2 Upstream Factor Contributes to Lung Ischemia‐Reperfusion Injury by Regulating Macrophage Polarization,” iScience 26, no. 9 (2023): 107496. 37636061 10.1016/j.isci.2023.107496 PMC10448077

[24] X. Gu , H. Kang , S. Cao , Z. Tong , and N. Song , “Blockade of TREM2 Ameliorates Pulmonary Inflammation and Fibrosis by Modulating Sphingolipid Metabolism,” Translational Research 275 (2025): 1–17. 39490681 10.1016/j.trsl.2024.10.002

[25] H. Cui , S. Banerjee , N. Xie , et al., “TREM2 Promotes Lung Fibrosis via Controlling Alveolar Macrophage Survival and Pro‐Fibrotic Activity,” Nature Communications 16, no. 1 (2025): 1761. 10.1038/s41467-025-57024-0 PMC11840137 39971937

[26] K. Wu , D. E. Byers , X. Jin , et al., “TREM‐2 Promotes Macrophage Survival and Lung Disease After Respiratory Viral Infection,” Journal of Experimental Medicine 212, no. 5 (2015): 681–697. 25897174 10.1084/jem.20141732 PMC4419356

[27] C. K. How , Y. Chien , K. Y. Yang , et al., “Induced Pluripotent Stem Cells Mediate the Release of Interferon Gamma‐Induced Protein 10 and Alleviate Bleomycin‐Induced Lung Inflammation and Fibrosis,” Shock 39, no. 3 (2013): 261–270. 23364435 10.1097/SHK.0b013e318285f2e2

[28] W. K. Yu , Y. P. Yang , W. C. Chen , et al., “Induced Pluripotent Stem Cell‐Derived Conditioned Medium, as Well as Nintedanib, Ameliorates Bleomycin‐Induced Pulmonary Fibrosis via Suppressing Endothelial‐Mesenchymal Transition,” Respiratory Investigation 63, no. 5 (2025): 904–914. 40683174 10.1016/j.resinv.2025.07.005

[29] S. D. Scoville , B. L. Mundy‐Bosse , M. H. Zhang , et al., “A Progenitor Cell Expressing Transcription Factor RORγt Generates All Human Innate Lymphoid Cell Subsets,” Immunity 44, no. 5 (2016): 1140–1150. 27178467 10.1016/j.immuni.2016.04.007 PMC4893782

[30] H. Furuya , Y. Toda , A. Iwata , et al., “Stage‐Specific GATA3 Induction Promotes ILC2 Development After Lineage Commitment,” Nature Communications 15, no. 1 (2024): 5610. 10.1038/s41467-024-49881-y PMC11226602 38969652

[31] S. Cording , J. Medvedovic , M. Cherrier , and G. Eberl , “Development and Regulation of RORγt+ Innate Lymphoid Cells,” FEBS Letters 588, no. 22 (2014): 4176–4181. 24681095 10.1016/j.febslet.2014.03.034

[32] H.‐c. Yao , Y. Zhu , H.‐y. Lu , et al., “Type 2 Innate Lymphoid Cell‐Derived Amphiregulin Regulates Type II Alveolar Epithelial Cell Transdifferentiation in a Mouse Model of Bronchopulmonary Dysplasia,” International Immunopharmacology 122 (2023): 110672. 37480752 10.1016/j.intimp.2023.110672

[33] A. Esparza‐Baquer , I. Labiano , O. Sharif , et al., “TREM‐2 Defends the Liver Against Hepatocellular Carcinoma Through Multifactorial Protective Mechanisms,” Gut 70, no. 7 (2021): 1345–1361. 32907830 10.1136/gutjnl-2019-319227 PMC8223629

[34] T. Hendrikx , F. Porsch , M. G. Kiss , et al., “Soluble TREM2 Levels Reflect the Recruitment and Expansion of TREM2(+) Macrophages That Localize to Fibrotic Areas and Limit NASH,” Journal of Hepatology 77, no. 5 (2022): 1373–1385. 35750138 10.1016/j.jhep.2022.06.004

[35] S. Li , J. W. Bostick , J. Ye , et al., “Aryl Hydrocarbon Receptor Signaling Cell Intrinsically Inhibits Intestinal Group 2 Innate Lymphoid Cell Function,” Immunity 49, no. 5 (2018): 915–28.e5. 30446384 10.1016/j.immuni.2018.09.015 PMC6249058

[36] L. L. Mi , Y. Zhu , and H. Y. Lu , “A Crosstalk Between Type 2 Innate Lymphoid Cells and Alternative Macrophages in Lung Development and Lung Diseases (Review),” Molecular Medicine Reports 23, no. 6 (2021): 403. 33786611 10.3892/mmr.2021.12042 PMC8025469

[37] K. Takahashi , C. D. P. Rochford , and H. Neumann , “Clearance of Apoptotic Neurons Without Inflammation by Microglial Triggering Receptor Expressed on Myeloid Cells‐2,” Journal of Experimental Medicine 201, no. 4 (2005): 647–657. 15728241 10.1084/jem.20041611 PMC2213053

[38] T. Jiang , L. Tan , X.‐C. Zhu , et al., “Upregulation of TREM2 Ameliorates Neuropathology and Rescues Spatial Cognitive Impairment in a Transgenic Mouse Model of Alzheimer's Disease,” Neuropsychopharmacology 39, no. 13 (2014): 2949–2962. 25047746 10.1038/npp.2014.164 PMC4229581

[39] B. R. Price , T. L. Sudduth , E. M. Weekman , et al., “Therapeutic Trem2 Activation Ameliorates Amyloid‐Beta Deposition and Improves Cognition in the 5XFAD Model of Amyloid Deposition,” Journal of Neuroinflammation 17, no. 1 (2020): 238. 32795308 10.1186/s12974-020-01915-0 PMC7427742

[40] A. Schulz‐Kuhnt , V. Greif , K. Hildner , et al., “ILC2 Lung‐Homing in Cystic Fibrosis Patients: Functional Involvement of CCR6 and Impact on Respiratory Failure,” Frontiers in Immunology 11 (2020): 691. 32457736 10.3389/fimmu.2020.00691 PMC7221160

