# Supplementary material for: Triggering Receptor Expressed on Myeloid Cells‐2 Regulates Innate Lymphoid Cell Levels in Bleomycin‐Induced Pulmonary Fibrosis
Source: Kaohsiung J Med Sci. 2026 Apr 2;42(11):e70209. doi: 10.1002/kjm2.70209 (PMC13399741; doi:10.1002/kjm2.70209)
Supplement: Supplementary file 1 [file KJM2-42-e70209-s001.pdf]

Supplementary profile

Data S1

Lung injury score

For the assessment of acute lung injury severity using histological methods, a lung injury scoring system was implemented. Two separate evaluators, working independently and without prior knowledge of the specimen details, examined each slide stained with hematoxylin and eosin as well as by IHC. For determination of the extent of the pathological changes, a total of 300 alveoli per slide were examined under 400X magnification. Points were allocated within each field on the basis of a pre-established set of criteria from a previous study[1-3]: The lung injury score was calculated using the following formula: lung injury score = ([alveolar hemorrhage points/number of fields] + 2 × [alveolar infiltrate points/number of fields] + 3 × [fibrin points/number of fields] + [alveolar septal congestion/number of fields])/total number of alveoli counted.

Ashcroft scale for pulmonary fibrosis evaluation

Pulmonary fibrosis severity was assessed using the Ashcroft scale, a numerical scoring system ranging from 0 (normal lung) to 8 (complete fibrotic obliteration). Histological lung sections were stained with Masson's trichrome to highlight collagen deposition. Fibrosis scoring was performed by evaluating randomly selected microscopic fields under a 20× objective, following predefined criteria for each grade. To ensure consistency and minimize observer bias, all assessments were normalized using a modified Ashcroft scale with standardized definitions for intermediate grades (2, 4, 6) and a systematic scanning pattern to reduce variability[4].

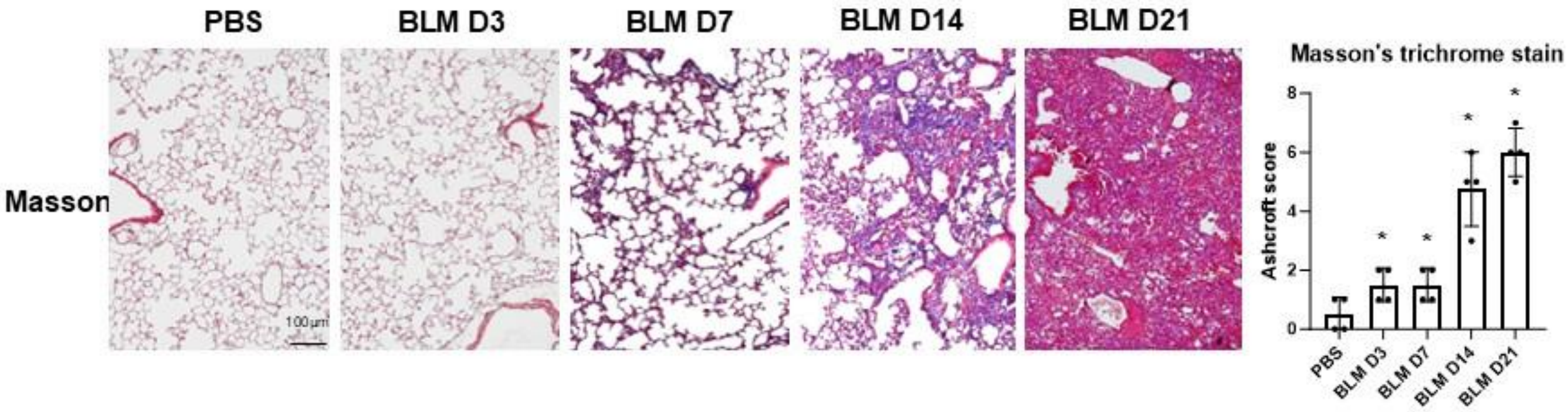

Figure S1. HE and Masson staining of mouse lung tissue following bleomycin treatment.

In C57BL/6 mice, a single intratracheal (i.t.) administration of bleomycin (BLM) led to a progressive course of lung injury and fibrosis. Histopathological assessment showed evident inflammatory damage as early as day 3, prominent interstitial fibrosis with collagen accumulation and tissue architecture disruption by day 14, and almost total loss of normal parenchymal structure by day 21. *$p < 0.05$ compared with the control group. The p-value was calculated using pairwise Student's t-tests. n=3–5 per group.

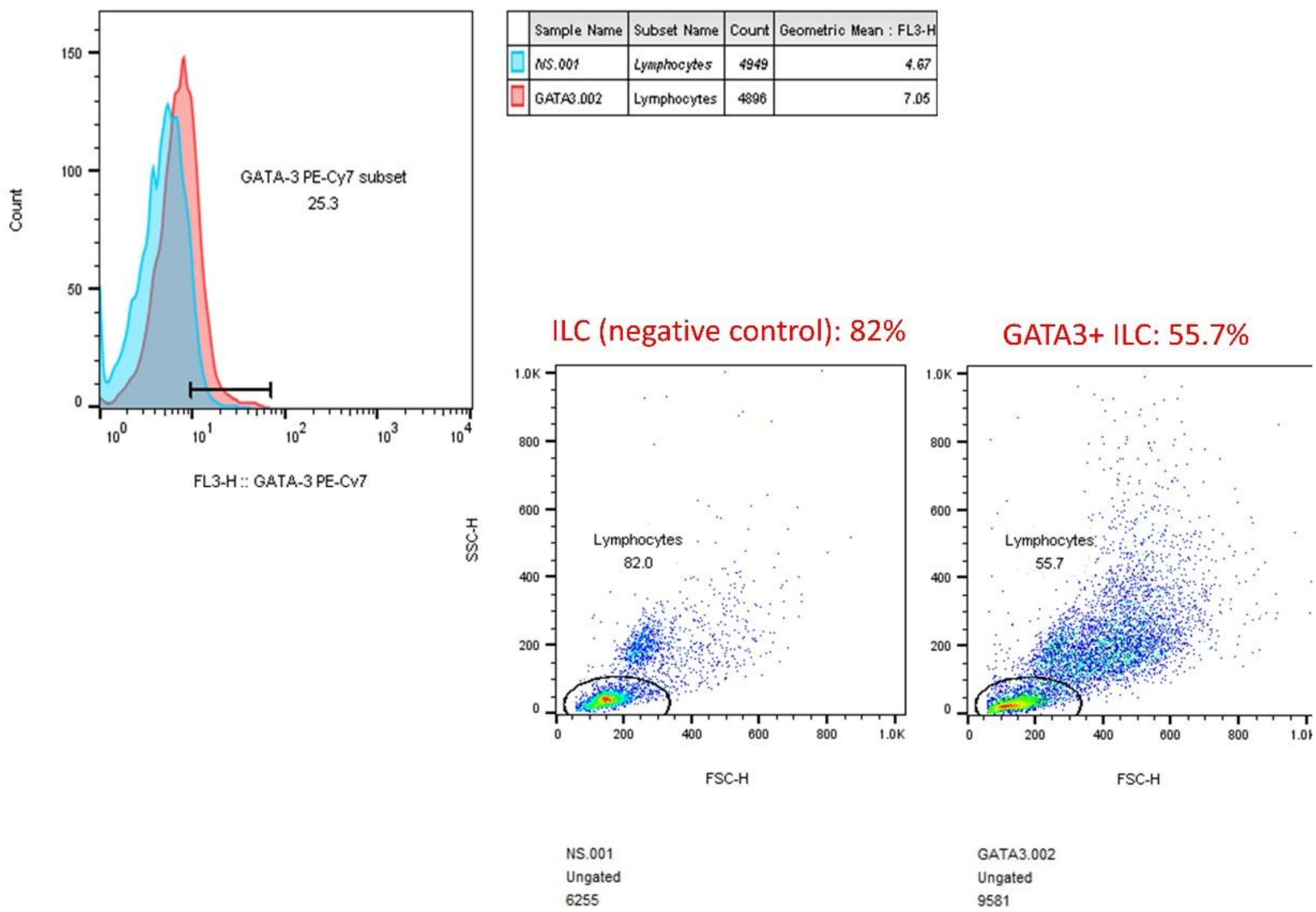

Figure S2. Flow cytometric characterization of MACS-isolated ILC-enriched population.

Cells were analyzed by flow cytometry after MACS isolation to verify ILC purity and subset composition. Lymphocytes were gated by FSC/SSC, showing that ~82% of events fell within the lymphocyte gate, confirming successful enrichment. Intracellular GATA3 staining identified ILC2s within

this population, with $GATA3^{+}$ cells accounting for 55.7% of isolated ILC-enriched population.

Reference

1. Matute-Bello G, Winn RK, Jonas M, Chi EY, Martin TR, Liles WC. Fas (CD95) induces alveolar epithelial cell apoptosis in vivo: implications for acute pulmonary inflammation. The American journal of pathology. 2001;158(1):153-61.
2. How CK, Chien Y, Yang KY, Shih HC, Juan CC, Yang YP, et al. Induced pluripotent stem cells mediate the release of interferon gamma-induced protein 10 and alleviate bleomycin-induced lung inflammation and fibrosis. Shock. 2013;39(3):261-70.
3. Su VY, Chiou SH, Lin CS, Mo MH, Yang KY. Induced Pluripotent Stem Cells Attenuate Endothelial Leakage in Acute Lung Injury via Tissue Inhibitor of Metalloproteinases-1 to Reduce Focal Adhesion Kinase Activity. Stem Cells. 2019;37(12):1516-27.
4. Hübner RH, Gitter W, El Mokhtari NE, Mathiak M, Both M, Bolte H, et al. Standardized quantification of pulmonary fibrosis in histological samples. Biotechniques. 2008;44(4):507-11, 14-7.
